# Is It Safe to Omit Any Chest X‐Ray Before Removing the Chest Drain After Elective, Non‐Cardiac Thoracic Surgery? A Single‐Center, Retrospective, Case–Control Study

**DOI:** 10.1111/1759-7714.70050

**Published:** 2025-03-27

**Authors:** Ioannis Karampinis, Carolin Reker, Laura Grifone, Fabio Souschek, Christian Galata, Davor Stamenovic, Eric Roessner

**Affiliations:** ^1^ Department of Thoracic Surgery, Center for Thoracic Diseases University Medical Center Mainz, Johannes Gutenberg University Mainz Mainz Germany; ^2^ Clinic for Diagnostic and Interventional Radiology University Medical Center Mainz, Johannes Gutenberg University Mainz Mainz Germany

**Keywords:** lobectomy, lung cancer, lung surgery, perioperative care, thoracic surgery, X‐ray

## Abstract

**Background:**

Every patient undergoing non‐cardiac thoracic surgery will receive several chest X‐rays through the perioperative period. The patient might receive a preoperative X‐ray as a baseline as well as several X‐rays before and after drain removal. This routine has several disadvantages, for the patient, the health care system and the medical staff. Purpose of this study was to examine if all X‐rays before removal of the drain can be omitted.

**Methods:**

Two hundred fifty‐five patients who underwent elective thoracic surgery were included in this retrospective analysis. Patients undergoing urgent procedures or empyema surgery, as well as patients with symptoms requiring further diagnostic measures or patients who required clamping of the drain before removal, were excluded.

**Results:**

Forty‐five patients received an X‐ray before removal of the drain, and 210 patients did not. The X‐ray group developed significantly more minor complications than the no X‐ray group. 46.7% of the X‐rays before drain removal (X‐ray group) were reported with abnormalities. However, these abnormalities never led to a change in patient care. Drainage time and postoperative hospital stay were significantly longer in the X‐ray group.

**Conclusions:**

Omitting any X‐ray between surgery and removal of the chest drain appears to be safe in our retrospective patient cohort. The proposed benefits of omitting the X‐ray are very relevant for the health care system, the medical and nursing teams, and, more importantly, for the patients. Evidence suggests that X‐ray of patients regularly do not exist. It is therefore reasonable to consider exploring this question in a formal prospective trial.

## Graphical abstract

summary of study design and results.

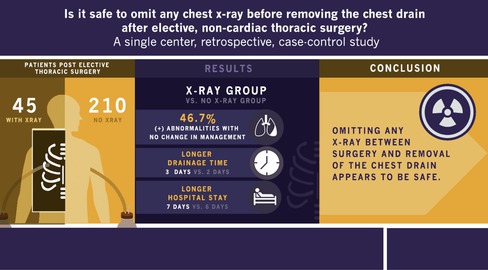

## 1 Introduction

In the perioperative setting of non‐cardiac thoracic surgery, the average patient will receive at least four or five X‐rays. The number of X‐rays mainly depends on the thoracic center and the established pathways. The first X‐ray is usually performed before surgery in order to ensure that a baseline image is available for later comparison. The second X‐ray normally follows the surgical procedure and is either performed in the recovery unit, intensive care unit, or on the ward. The rationale for this second X‐ray is to rule out immediate postoperative bleeding, check the positioning of the chest tubes or central venous lines, as well as the lung expansion following the procedure. A third X‐ray is normally performed on the first postoperative day, and several other X‐rays might be requested on the days that follow. Every patient will certainly receive an X‐ray after drain removal. This specific X‐ray serves several purposes, both clinical and medicolegal [1]. The last X‐ray typically comes with the follow‐up visit in the outpatient clinic.

Requesting X‐rays in the perioperative setting has several advantages, both for the patient and the surgeon. The main advantage for the patient is that a surgical complication might be detected earlier and before becoming clinically relevant. As for the surgeon, the X‐ray offers reassurance about the clinical condition of the patient, can be reviewed anytime and by different examiners, and does not require the surgeon to do anything other than review it. On the other hand, the evidence for performing all these X‐rays is very limited. Furthermore, X‐rays expose the patient to radiation, which adds to the radiation coming from the CT scan and potentially the PET scan that the patient might have had [2]. That leads to the cumulative exposure significantly exceeding the annual limit of 1 mSv. In addition to the exposure, X‐rays increase not only the treatment costs but also the work burden, since it usually takes a surgical trainee to request it, a secretary to schedule it, the radiographer to perform it, a radiological trainee and a consultant to report it, and the surgical team to review it. Last but not least, most thoracic surgery patients are cancer patients. These patients undergo multiple examinations and procedures throughout their treatment, each one adding stress and discomfort.

Our thoracic surgical department was newly established at the end of 2019. Following the COVID‐19 pandemic, we reviewed and updated our standard operating procedures based on the available evidence. With regard to perioperative X‐ray requests, we omit any X‐ray before removing the chest drain in patients who are asymptomatic and have not undergone urgent procedures. The purpose of this study was to review the results of the implementation of our new standard operating procedure (SOP).

## 2 Materials and Methods

The study was conducted in accordance with the Declaration of Helsinki and was approved by the local ethics committee (2021‐15 979). Patients were included in this retrospective analysis over a period of 18 months. Due to the fact that the implementation of a new SOP does not take place immediately, the patient cohort in this study includes both patients who did not have an X‐ray before the removal of the drain and patients who had an X‐ray before drain removal without having any particular indication to receive the X‐ray based on our standard of care standard operating procedure. Only patients where the indication on the requesting form of the X‐ray was just “postoperative control” or other synonymous terms were included.

The following patients were excluded from the study:

- urgent thoracic procedures,
- pediatric patients,
- patients who had more than one X‐ray before removing the drain,
- patients undergoing empyema surgery,
- drain clamping before removal, and
- patients with any symptoms requiring further diagnostics.

These exclusion criteria reflect the recommendations of our internal standard operating procedure. The primary endpoint of the study was to compare the complication rate between the group of patients without an X‐ray before the removal of the drain and the patients who had an X‐ray before the removal of the drain. Reporting of postoperative complications was completed after the patient had been evaluated in the outpatient clinic, usually 10–14 days after discharge. All patients, both the X‐ray and the no X‐ray group, received an X‐ray during this visit. Postoperative complications that led to readmission were reported regardless of the time point. The systematic classification of morbidity and mortality after thoracic surgery was used to categorize complications [3]. Major morbidity was defined as complication grade ≥ 3. Since this is a retrospective study, in‐hospital mortality was used to express mortality rates.

### 2.1 Data Analysis

Quantitative variables are presented as median with interquartile range (IQR). Bivariate analysis of categorical variables was performed using two‐tailed χ^2^ or the Fisher's exact test. 5% was used as a cut‐off for statistical significance. The statistical analysis was performed using GraphPad Prism (Version 9.0.1 for Windows, GraphPad Software, San Diego, California USA, www.graphpad.com).

## 3 Results

Two hundred fifty‐five patients were included in this study. Forty‐five patients had received an X‐ray before the removal of the drain (X‐ray group) and 210 patients had their drain removed without having had any X‐ray between surgery and drain removal (no X‐ray group). The baseline characteristics of the two groups were comparable in terms of demographics, BMI, ASA score (ASA: American Society of Anesthesiologists), and preoperative forced expiratory volume in 1 s. The number of major lung resections in the two groups was also comparable (Table 1).

**TABLE 1:** Patient characteristics.

	X‐ray	No X‐ray	p‐value
Age	66 (IQR 13.5)	65 (IQR 18)	0.72
BMI	26.6 (IQR 6.9)	26.60 (IQR 6.7)	0.39
ASA Score (2/3/4)	11/30/2	56/129/20	n.s.
ALR	12 (26.7%)	61 (29%)	0.85
VATS	26 (57.8%)	157 (74.8%)	0.02
VATS–ALR	6 (50%)	40 (70.2%)	0.19
FEV1 (%)	81 (IQR 25.9)	84 (IQR 30)	0.28
Drainage time	3 (median)	2 (median)	< 0.001
Length of stay	7 (median)	6 (median)	< 0.001

*Note:* Expressed as percentage of the predictive value.

Abbreviations: *BMI: body mass index kg/m* ^*2*^, *ASA: American Society of Anesthesiologists, IQR*: *interquartile range, ALR: anatomic lung resections, VATS: video‐assisted thoracic surgery, FEV1 (%): forced expiratory volume in 1 s, expressed as percentage of the predictive value*.

All drains used in our department are soft, silicone drains with several fenestrations. Thirty‐eight patients of the X‐ray group received a single 18 French drain after surgery, 2 patients had a 20 French drain, and one patient had a single 24 French drain. One hundred forty‐three patients of the no X‐ray group had a single 18 French drain, one patient received two 18 French drains, and 8 patients had a 20 French drain.

The interval between X‐ray and drain removal was 2 days (IQR 2) for the X‐ray group. The radiologists described pathological findings in 21 of 45 patients who received an X‐ray before the removal of the chest drain. The X‐ray findings did not change patient care in any of these cases.

### 3.1 Postoperative Complications

Fifteen patients of the X‐ray group and 41 patients of the no X‐ray group developed postoperative complications. The overall complication rate was significantly higher in the X‐ray group (Table 2).

**TABLE 2:** Postoperative complications [3].

Complications	X‐ray	No X‐ray	*p*‐value
Any	15/45 (33.3%)	41/210 (19.5%)	0.048
Grade I	3/45 (6.67%)	28/210 (13.3%)	0.31
Pneumothorax/dead space	2	17	
Pleural effusion	1	4	
Wound healing disorders	0	3	
Subcut. emphysema	0	4	
Grade II	8/45 (17.78%)	10/210 (4.76%)	0.005
Pneumonia	2	5	
Prolonged air leak	2	2	
Wound infection	0	2	
Respir. Failure	0	1	
Pleural effusion	4	0	
Grade IIIa	2/45 (4.45%)	0/210	0.03
Hemoptysis	1	0	
Recurr. Pleural effusion	1	0	
Grade IIIb	2/45 (4.45%)	3/210 (1.42%)	0.14
Wound infection	1	0	
Pneumothorax	1	0	
Retained chest drain	0	1	
Pericardial effusion	0	1	
Pleural effusion	0	1	

**FIGURE 1:**
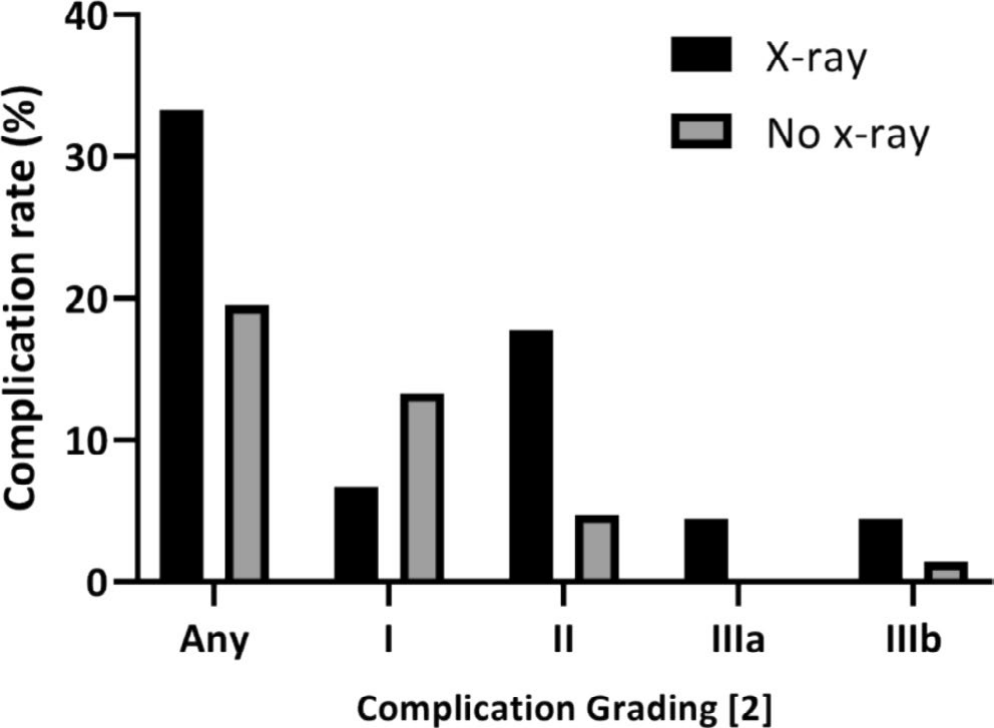
Postoperative complication rate (%).

The rate of grade II complications and the rate of grade IIIa complications were also significantly higher in the X‐ray group, while the rate of grade IIIb complications (surgical revisions) was not different between the two groups (Figure 1).

**FIGURE 2:**
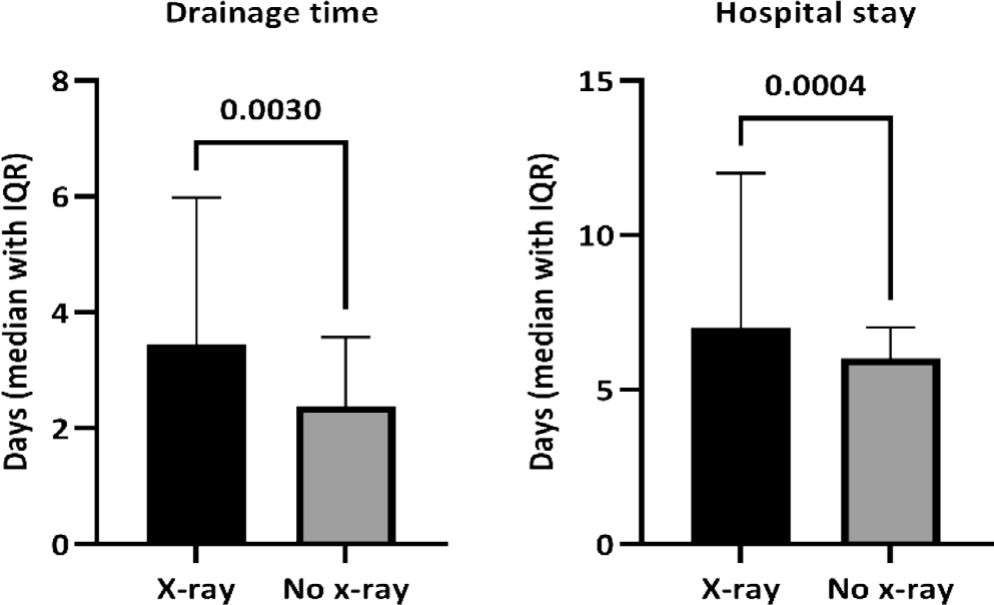
Postoperative drainage time and length of hospital stay.

The median length of drainage time was significantly longer for the X‐ray group compared with the no X‐ray group (3 days vs. 2 days, *p* < 0.001). Consistently, the postoperative hospital stay was also significantly longer in the X‐ray group compared with the no X‐ray group (7 days vs. 6 days, p < 0.001, Figure 2).

### 3.2 Subgroup Analysis

In the group of patients that underwent anatomic lung resections, we identified four patients from the X‐ray group that developed grade I complications (33.3%). Fourteen patients of the no‐X‐ray group developed a grade I complication (6.67%). Furthermore, five patients of the no X‐ray group and three patients of the no X‐ray group developed grade II and grade IIIb complications, respectively. The revisional surgical procedures were a redo thoracoscopy with the removal of a dislodged drain and a redo thoracoscopy with the insertion of an indwelling pleural catheter due to recurring symptomatic pleural effusion.

## 4 Discussion

The undoubtedly great value of chest X‐rays in the field of thoracic surgery was first described over 70 years ago [4]. Since then, X‐rays have been regularly performed in the perioperative window of thoracic surgery. The main reason for that is that they can offer a significant amount of information about the lungs and the mediastinum, support the diagnosis of postoperative complications, and potentially point out complications before they become clinically significant.

The field of thoracic surgery has evolved significantly over the past two decades. The swift from open toward minimally invasive surgery, the introduction of modern anesthetic techniques and all the other aspects of the ERAS concept have contributed to the whole field of thoracic surgery becoming less invasive [5]. Our patients are no longer routinely equipped with central lines and arterial lines, the number of routine chest drains has been reduced down to one, and most clinics do not monitor their patients on a high‐dependency unit after the operation. The most interesting part of this “step down process” of intraoperative and postoperative care is that it does not compromise patient safety, spares manpower, time and resources and improves outcome, comfort, and patient experience.

Based on the general trend to reduce unnecessary measures in our field, we considered reducing the number of perioperative X‐rays down to the necessary ones. A systematic review that we performed last year showed us that there is not really solid evidence to support routine X‐rays in the perioperative window [1]. The findings of this study led us to update our standard operating procedure on X‐rays after thoracic surgery. According to our SOP, X‐rays are only performed in patients where a clinical concern indicates the X‐ray (unexpected hypoxia, elevated inflammatory markers, unexplained drop of hemoglobin values, etc.), in empyema patients where we need to assess the lung expansion, after the removal of the chest drain, and in the outpatient clinic. All other X‐rays before or after surgery are no longer performed.

In this study, we reviewed the results of this standard operating procedure. In the group of patients who received an X‐ray before the removal of the drain, almost half of the X‐rays were reported as pathological. That is probably the reason why this group had significantly more minor postoperative complications compared with the no X‐ray group. In this study, we defined as “pathological” every deviation from a completely normal postoperative X‐ray, including postoperative pleural effusions, any pneumothorax/dead space over 2 cm, as well as any opacifications on the X‐ray. It is certainly difficult to standardize what is or is not normal on a postoperative X‐ray, especially in a retrospective study. However, the X‐ray group patients received overall one X‐ray more than the no x‐ray group (3 vs. 2 X‐rays in total, *p* < 0.001) and this extra X‐ray never led to any change in the postoperative patient care. Even in the subgroup of patients with major lung resections who did not have an X‐ray before the removal of the drain, there were no incidences of patients requiring additional treatment measures compared to the group of patients who had an X‐ray before removal of the drain.

The results of our study agree with other studies that have been previously published in this field [6, 7, 8, 9]. These studies have questioned the indication of performing X‐rays on the day of surgery, every day following surgery, or after removing the chest tube. However, we have to keep in mind that in order to be able to solidify this new practice, rock‐hard evidence is required, and that can only be achieved through randomized controlled trials.

### 4.1 Limitations

The collection of the data in this study was performed prospectively, but the interpretation and processing were performed retrospectively and are therefore subject to all sorts of bias that retrospective studies are susceptible to. However, despite the retrospective nature of the study, all relevant information was available. Furthermore, this is a large cohort of patients with different diseases, surgical procedures performed, comorbidities of each patient, etc., which makes the sample heterogeneous and therefore limits the generalizability and strength of the conclusions.

## 5 Conclusion

Omitting any X‐ray before the removal of the drain appears to be safe, saves time and money and allows the patient to get rid of the drain and all the drain‐associated issues 1 day earlier and also to be discharged 1 day earlier. Saving 1 day of hospital stay is extremely difficult to achieve in modern thoracic surgery, and there are very few measures that have had such a major effect on reducing hospital stay. It might appear radical to propose to stop X‐raying patients before removing the drain, but we have to take into account that we are now collecting evidence supporting this pathway while we do not have any evidence suggesting to act differently. Considering the aforementioned benefits of the no X‐ray tactic, it is certainly worthwhile to explore this option in a formal prospective trial.

## Author Contributions

I.K. conceived of the presented idea, collected and analyzed the data, and drafted the manuscript. C.R. collected and analyzed the data. L.G. collected and analyzed the data. F.S. collected and analyzed the data. C.G. conceived the presented idea, collected, and analyzed the data. D.S. has reviewed the manuscript for important intellectual content. E.R. conceived of the presented idea and reviewed the manuscript for important intellectual content.

## Ethics Statement

The study was conducted in accordance with the Declaration of Helsinki and was approved by the local ethics committee (2021‐1979). Due to the retrospective nature of the study, no written informed consent was obtained.

## Consent

The authors have nothing to report.

## Conflicts of Interest

The authors declare no conflicts of interest.

## Data Availability

The datasets generated during the current study are not publicly available since the main part is included in this article. The complete database is available from the corresponding author upon reasonable request.
